# Supplementary material for: Evaluation of the Sensititre YeastOne and Etest in Comparison with CLSI M38-A2 for Antifungal Susceptibility Testing of Three Azoles, Amphotericin B, Caspofungin, and Anidulafungin, against Aspergillus fumigatus and Other Species, Using New Clinical Breakpoints and Epidemiological Cutoff Values
Source: Pharmaceutics. 2022 Oct 11;14(10):2161. doi: 10.3390/pharmaceutics14102161 (PMC9607534; doi:10.3390/pharmaceutics14102161)
Supplement: Supplementary file 1 [file pharmaceutics-14-02161-s001.zip › pharmaceutics-1873683-supplementary.pdf]

Supplementary Material: Table S1. Database on 106 clinical *Aspergillus* spp. isolates according to MIC (mg/L) results using two commercial methods and CLSI broth mibrodiution method

Table S1- Database on 106 clinical *Aspergillus* spp. isolates according to MIC (mg/L) results using two commercial methods and CLSI broth mibrodiution method

Isolates	Specie	BMD-CLSI posa	BMD-CLSI vcz	BMD-CLSI itc	BMD-CLSI amb	BMD-CLSI cas	BMD-CLSI anidu	Etest vcz	Etest itc	Etest caspo	Etest amb	Etest anidu	SYO pos	SYO vcz	SYO itc	SYO amb	SYO cas	SYO anidu
1	*A. fumigatus*	0,12	0,5	2	0,5	0,25	ND	ND	0,5	ND	0,25	ND	0,12	0,25	0,12	2	NR	NR
2	*A. niger*	0,5	0,5	0,06	0,5	0,25	0,03	0,125	0,38	0,023	0,032	0,002	ND	ND	ND	ND	ND	ND
3	*A. fumigatus*	0,12	0,5	0,5	2	0,25	0,001	0,25	0,5	0,032	1	0,002	0,12	0,5	0,25	2	NR	NR
4	*A. fumigatus*	0,12	0,5	0,5	2	0,25	0,015	0,75	0,5	ND	0,75	0,002	0,06	0,5	0,12	4	NR	NR
5	*A. fumigatus*	0,12	0,5	0,12	0,5	0,5	ND	0,125	0,75	0,012	6	ND	ND	ND	ND	ND	ND	ND
6	*A. fumigatus*	0,12	0,5	1	0,5	0,25	0,03	0,25	2	0,094	0,38	ND	0,12	0,5	0,5	2	NR	NR
7	*A. fumigatus*	0,25	0,5	0,25	0,25	0,25	ND	0,38	6	ND	0,38	ND	0,25	2	0,5	1	NR	NR
8	*A. fumigatus*	0,06	1	2	0,5	0,12	ND	ND	ND	ND	0,25	ND	0,25	0,5	0,25	2	NR	NR
9	*A. fumigatus*	0,12	0,5	0,5	0,5	0,5	0,003	0,125	1	ND	0,5	ND	0,5	0,5	0,06	0,5	0,25	0,003
10	*A. fumigatus*	0,12	0,5	0,5	0,5	0,12	ND	ND	ND	ND	0,002	ND	0,12	0,25	0,25	1	NR	NR
11	*A. fumigatus*	0,25	0,5	1	0,5	0,25	ND	ND	1	0,047	0,19	ND	ND	ND	ND	ND	NR	ND
12	*A. fumigatus*	0,12	1	0,5	0,5	0,5	ND	ND	0,94	0,19	0,38	ND	ND	ND	ND	ND	NR	ND
13	*A. fumigatus*	0,25	0,5	0,25	1	0,5	ND	0,19	0,06	0,094	1,5	ND	0,12	0,5	0,25	2	NR	NR
14	*A. fumigatus*	0,12	0,25	0,06	0,5	0,25	ND	ND	0,06	ND	0,047	ND	ND	ND	ND	ND	ND	ND
15	*A. fumigatus*	0,12	0,125	0,5	0,25	0,5	0,003	0,125	1	ND	0,002	0,002	0,5	1	0,5	1	NR	NR
16	*A. flavus*	0,12	0,5	0,5	0,5	0,12	ND	ND	ND	ND	0,19	ND	0,12	0,5	0,25	2	NR	NR
17	*A. fumigatus*	0,12	0,5	2	2	0,25	0,003	1	1,5	ND	0,75	0,002	0,25	0,12	8	8	0,008	0,015
18	*A. fumigatus*	0,12	0,5	1	2	0,5	0,003	0,19	0,5	ND	1,5	0,003	0,12	0,12	0,12	2	NR	NR
19	*A. fumigatus*	0,25	0,5	0,5	1	0,5	ND	0,19	0,75	0,032	0,5	ND	0,12	0,5	0,25	2	NR	NR
20	*A. fumigatus*	0,12	0,25	0,5	0,5	1	0,007	1,5	1	ND	0,5	0,002	0,5	0,5	0,25	1	NR	NR
21	*A. fumigatus*	0,12	ND	1	0,25	0,5	ND	ND	0,125	ND	0,064	ND	ND	ND	ND	ND	ND	ND
22	*A. fumigatus*	0,12	0,5	1	2	0,5	0,003	0,125	1	ND	0,002	0,002	ND	ND	ND	ND	ND	ND
23	*A. fumigatus*	0,12	0,5	0,25	0,5	0,25	ND	ND	ND	ND	0,25	ND	ND	ND	ND	ND	ND	ND
24	*A. fumigatus*	0,12	0,5	1	1	0,5	ND	ND	1	ND	0,004	ND	0,06	0,25	0,25	1	NR	NR
25	*A. fumigatus*	0,12	0,25	0,12	0,25	0,25	ND	0,19	0,5	0,002	0,38	ND	ND	ND	ND	ND	ND	ND
26	*A. fumigatus*	0,25	0,5	0,12	1	0,25	0,003	0,125	0,5	0,064	0,75	ND	0,12	0,5	0,25	2	NR	NR
27	*A. flavus*	0,12	0,25	0,12	2	0,5	0,015	0,125	1,5	ND	32	0,02	0,12	0,25	0,12	1	0,5	0,06
28	*A. flavus*	0,12	0,5	0,06	2	0,25	0,003	0,25	0,5	ND	1	0,02	0,12	0,25	0,5	2	NR	NR
29	*A. fumigatus*	0,12	1	0,5	0,5	0,5	ND	0,38	1	ND	0,19	ND	ND	ND	ND	ND	ND	ND
30	*A. fumigatus*	0,12	0,5	0,5	0,25	0,25	ND	0,19	2	0,094	0,25	ND	ND	ND	ND	ND	ND	ND
31	*A. fumigatus*	0,12	0,25	0,5	0,5	0,5	ND	0,125	0,75	0,125	0,047	ND	ND	ND	ND	ND	ND	ND
32	*A. fumigatus*	0,5	1	8	0,5	0,12	ND	0,25	0,75	0,094	0,125	ND	ND	ND	ND	ND	ND	ND
33	*A. fumigatus*	0,25	2	0,06	1	0,25	0,015	0,75	0,5	0,125	0,094	0,002	0,5	2	0,5	2	NR	NR
34	*A. fumigatus*	0,12	0,5	0,25	0,5	0,5	ND	ND	ND	ND	0,25	ND	ND	ND	ND	ND	ND	ND
35	*A. fumigatus*	0,25	1	0,25	0,5	0,5	ND	0,19	0,75	ND	3	ND	ND	ND	ND	ND	ND	ND
36	*A. niger*	0,25	0,5	1	0,25	0,25	ND	0,25	2	ND	0,38	ND	ND	ND	ND	ND	ND	ND
37	*A. niger*	0,12	0,5	1	0,5	0,25	ND	ND	ND	ND	0,38	ND	ND	ND	ND	ND	ND	ND
38	*A. fumigatus*	0,12	0,5	1	0,5	0,5	ND	0,125	0,75	ND	0,016	ND	ND	ND	ND	ND	ND	ND
39	*A. fumigatus*	0,12	0,25	2	1	0,5	0,001	0,09	1,5	ND	0,19	ND	0,25	0,5	0,12	2	NR	NR
40	*A. fumigatus*	0,06	0,5	1	1	0,5	ND	ND	ND	ND	1,5	ND	0,03	0,25	0,06	1	NR	NR
41	*A. fumigatus*	0,25	1	1	2	0,5	0,003	0,125	0,38	ND	2	ND	0,12	0,5	0,25	2	NR	NR
42	*A. fumigatus*	0,12	0,5	1	2	0,5	ND	0,25	0,5	ND	0,023	ND	ND	ND	ND	ND	ND	ND
43	*A. fumigatus*	0,12	0,5	1	1	0,25	ND	0,19	1	0,002	4	ND	ND	ND	ND	ND	ND	ND
44	*A. fumigatus*	0,12	0,5	0,25	0,5	0,25	ND	ND	ND	ND	0,19	ND	ND	ND	ND	ND	ND	ND
45	*A. fumigatus*	0,12	1	0,5	0,5	0,25	ND	ND	ND	ND	0,006	ND	ND	ND	ND	ND	ND	ND
46	*A. fumigatus*	0,12	1	0,5	1	0,25	ND	0,19	1,5	0,047	2	ND	ND	ND	ND	ND	ND	ND
47	*A. clavatus*	0,12	2	1	1	0,5	0,003	1	1	ND	0,004	0,002	0,5	1	0,5	1	NR	NR
48	*A. fumigatus*	0,12	1	1	0,5	0,5	ND	ND	ND	ND	0,38	ND	ND	ND	ND	ND	ND	ND
49	*A. fumigatus*	0,12	0,5	1	0,25	0,25	ND	0,19	0,5	0,002	0,38	ND	ND	ND	ND	ND	ND	ND
50	*A. fumigatus*	0,12	0,25	0,25	0,5	0,5	0,015	0,38	1	0,125	0,002	0,003	0,12	0,5	0,5	1	NR	NR
51	*A. fumigatus*	0,12	0,5	1	0,25	0,5	ND	ND	ND	ND	0,19	ND	ND	ND	ND	ND	ND	ND
52	*A. awamori*	0,25	0,5	1	0,3	1	ND	ND	ND	0,032	0,19	ND	ND	ND	ND	ND	ND	ND
53	*A. fumigatus*	0,12	0,5	0,25	1	0,5	ND	0,19	1	0,125	0,38	ND	1	0,5	0,25	8	0,03	0,015
54	*A. fumigatus*	0,25	1	1	1	0,5	0,003	0,75	1	ND	0,25	0,002	ND	ND	ND	ND	ND	ND
55	*A. clavatus*	0,12	2	0,5	1	0,5	0,003	1	0,5	0,094	0,5	0,002	ND	ND	ND	ND	ND	ND
56	*A. flavus*	0,12	0,5	0,25	0,5	0,25	ND	ND	0,75	ND	0,75	ND	0,12	0,5	0,25	2	NR	NR
57	*A. fumigatus*	0,12	0,5	0,5	0,5	0,5	ND	ND	ND	ND	0,25	ND	ND	ND	ND	ND	ND	ND
58	*A. fumigatus*	0,12	0,125	0,25	1	0,5	ND	ND	2	ND	0,38	ND	0,25	0,25	0,25	8	0,008	0,015
59	*A. fumigatus*	0,12	0,5	1	1	0,5	ND	0,25	2	0,064	0,125	ND	0,06	4	8	8	1	0,03
60	*A. fumigatus*	0,06	0,5	0,25	1	0,5	ND	ND	1,5	ND	1	ND	0,25	0,25	0,25	1	0,12	0,015
61	*A. fumigatus*	0,12	0,5	0,5	1	1	ND	ND	ND	0,064	0,38	ND	0,12	0,5	0,25	2	NR	NR
62	*A. niger*	0,12	0,5	0,5	0,5	0,5	ND	ND	ND	ND	1	ND	ND	ND	ND	ND	ND	ND
63	*A. fumigatus*	0,5	0,5	0,25	0,5	0,5	ND	ND	ND	ND	0,25	ND	0,5	1	0,5	2	NR	NR
64	*A. fumigatus*	0,25	0,5	0,25	1	1	0,015	0,19	1,5	0,047	0,25	0,002	0,12	1	0,5	1	0,25	0,015
65	*A. terreus*	0,12	1	0,5	1	0,5	0,015	0,19	1	0,094	1	0,003	ND	ND	ND	ND	ND	ND
66	*A. fumigatus*	0,12	0,5	1	1	0,5	ND	ND	ND	ND	0,064	0,003	ND	ND	ND	ND	ND	ND
67	*A. fumigatus*	0,12	0,5	0,5	0,5	0,25	0,003	0,125	1	0,064	0,75	0,003	ND	ND	ND	ND	ND	ND
68	*A. welwitschiae*	0,12	0,5	1	1	0,5	ND	0,125	0,75	0,064	0,38	ND	0,12	0,25	0,5	2	NR	NR
69	*A. fumigatus*	0,25	0,5	0,06	0,5	0,5	ND	0,19	1	ND	1	ND	0,12	0,5	0,5	2	NR	NR
70	*A. fumigatus*	0,12	0,5	0,5	1	0,5	0,03	0,25	0,75	0,047	0,75	0,002	ND	ND	ND	ND	ND	ND
71	*A. fumigatus*	0,12	0,25	0,5	0,3	0,5	ND	ND	ND	ND	0,094	ND	ND	ND	ND	ND	ND	ND
72	*A. niger*	0,25	0,25	0,5	1	0,5	ND	ND	1,5	ND	0,002	ND	0,25	0,5	0,5	2	NR	NR
73	*A. clavatus*	0,12	2	0,25	0,5	0,25	ND	1,5	0,5	0,047	0,5	0,002	0,12	0,5	0,25	2	NR	NR
74	*A. flavus*	0,06	0,5	0,5	0,25	0,5	ND	ND	ND	ND	0,19	ND	0,12	0,5	0,5	2	NR	NR
75	*A. fumigatus*	0,12	0,5	2	1	1	0,015	0,25	0,75	0,047	1,5	0,002	0,12	0,5	0,25	2	NR	NR
76	*A. fumigatus*	0,5	0,5	0,5	1	1	ND	0,19	0,5	0,032	0,38	ND	0,12	0,5	0,25	2	NR	NR
77	*A. fumigatus*	0,5	0,25	1	1	0,5	0,015	0,19	0,5	0,094	0,25	0,002	0,06	0,12	0,25	2	NR	NR
78	*A. fumigatus*	1	1	0,5	2	0,5	0,03	0,125	1,5	0,064	1	0,002	0,12	0,25	0,25	1	NR	NR
79	*A. fumigatus*	0,12	1	1	0,5	0,5	ND	0,19	1	0,047	0,002	ND	ND	ND	ND	ND	ND	ND
80	*A. fumigatus*	0,12	0,25	0,5	1	0,12	ND	ND	ND	ND	0,75	ND	0,12	0,5	0,25	2	NR	NR
81	*A. flavus*	0,25	ND	0,12	0,5	0,5	0,015	0,125	1	ND	0,38	0,002	0,12	0,5	0,5	2	NR	NR
82	*A. niger*	0,12	0,5	0,25	0,5	0,5	0,003	0,25	2	0,125	1,5	0,002	ND	ND	ND	ND	ND	ND
83	*A. fumigatus*	0,25	0,5	0,5	1	0,5	ND	ND	ND	ND	1	ND	0,25	1	0,5	2	NR	NR
84	*A. fumigatus*	0,12	1	1	2	0,25	0,003	0,25	0,75	0,047	1,5	0,002	0,12	0,5	0,25	2	NR	NR
85	*A. fumigatus*	0,25	0,5	0,5	0,5	0,5	ND	0,25	1	ND	0,002	ND	0,12	0,5	0,5	2	NR	NR
86	*A. fumigatus*	0,12	0,5	1	1	0,25	0,001	0,19	1	0,032	8	0,002	0,12	0,5	0,5	2	NR	NR
87	*A. fumigatus*	0,12	0,5	0,5	0,5	0,5	ND	0,5	1	ND	0,5	0,004	0,25	0,5	0,25	2	NR	NR
88	*A. fumigatus*	0,12	0,5	0,25	1	0,5	ND	ND	0,5	ND	0,004	ND	0,12	0,5	0,25	2	NR	NR
89	*A. niger*	0,12	0,5	1	1	0,25	ND	ND	1,5	ND	0,25	ND	0,25	1	0,5	2	NR	NR
90	*A. niger*	0,25	0,5	1	1	1	ND	ND	ND	0,047	1	ND	ND	ND	ND	ND	ND	ND
91	*A. fumigatus*	0,12	1	0,25	0,5	0,25	ND	0,25	1,5	ND	0,094	ND	ND	ND	ND	ND	ND	ND
92	*A. fumigatus*	0,12	0,25	1	0,5	0,25	ND	0,19	1	ND	0,38	ND	ND	ND	ND	ND	ND	ND
93	*A. fumigatus*	0,12	0,5	0,5	0,5	0,5	ND	ND	ND	ND	0,75	ND	ND	ND	ND	ND	ND	ND
94	*A. fumigatus*	0,12	0,5	0,25	2	0,5	0,007	0,19	1	0,125	0,75	0,004	0,25	0,5	0,25	2	NR	NR
95	*A. fumigatus*	0,12	0,5	0,25	0,5	0,25	ND	ND	ND	ND	0,5	ND	0,12	0,5	0,25	2	NR	NR
96	*A. niger*	0,12	1	1	0,5	0,25	ND	ND	ND	ND	0,5	ND	ND	ND	ND	ND	ND	ND
97	*A. fumigatus*	0,25	0,5	1	0,5	0,25	0,03	0,25	1	ND	0,125	0,002	0,5	1	0,5	2	NR	NR
98	*A. fumigatus*	0,12	0,25	0,75	0,5	0,12	ND	0,19	0,75	ND	0,38	ND	0,25	0,5	0,5	2	NR	NR
99	*A. fumigatus*	0,12	ND	0,5	0,5	1	ND	ND	ND	0,023	0,094	ND	ND	ND	ND	ND	ND	ND
100	*A. fumigatus*	0,12	0,25	0,25	0,5	0,25	ND	ND	ND	ND	0,5	ND	0,12	0,5	0,25	2	NR	NR
101	*A. fumigatus*	0,25	0,25	0,5	2	0,25	0,003	0,25	0,5	ND	0,5	0,002	ND	ND	ND	ND	ND	ND
102	*A. fumigatus*	0,25	0,25	0,5	1	0,5	ND	0,125	0,75	ND	0,75	ND	ND	ND	ND	ND	ND	ND
103	*A. flavus*	0,25	1	0,5	2	0,5	ND	0,75	0,5	ND	0,75	0,002	0,12	0,5	0,25	2	NR	NR
104	*A. fumigatus*	0,25	0,5	0,5	0,5	0,25	ND	0,19	1	0,125	0,38	ND	0,12	0,5	0,25	2	NR	NR
105	*A. fumigatus*	0,12	1	0,5	1	0,5	ND	0,19	0,75	0,19	2	ND	ND	ND	ND	ND	ND	ND
106	*A. fumigatus*	0,12	0,5	0,12	1	0,5	ND	0,25	1,5	0,125	0,5	0,002	ND	ND	ND	ND	ND	ND

SUBTITLES:
Isolates Number
BMD-CLSI: Broth microdilution by CLSI method
SYO: Sensitire YeastOne
posa: posaconazole
vcz: voriconazole
itc: itraconazole
amb: amphotericin B
cas: caspofungin
anidu: anidulafungin
NR: Values ≥ 8 not registered (NR), as informed in Material and Methods. Section 3.2.3 SYO.
ND: not done
